# Zinc Oxide-Based Nanomaterials for Microbiostatic Activities: A Review

**DOI:** 10.3390/jfb15040103

**Published:** 2024-04-15

**Authors:** Alemtsehay Tesfay Reda, Jae Yeon Park, Yong Tae Park

**Affiliations:** Department of Mechanical Engineering, Myongji University, 116 Myongji-ro, Cheoin-gu, Yongin, Gyeonggi 17058, Republic of Korea; atesfayreda@gmail.com (A.T.R.);

**Keywords:** Zinc oxide, nano-materials, antimicrobial, microbiostatic activity, nanotechnology

## Abstract

The world is fighting infectious diseases. Therefore, effective antimicrobials are required to prevent the spread of microbes and protect human health. Zinc oxide (ZnO) nano-materials are known for their antimicrobial activities. Because of their distinctive physical and chemical characteristics, they can be used in medical and environmental applications. ZnO-based composites are among the leading sources of antimicrobial research. They are effective at killing (microbicidal) and inhibiting the growth (microbiostatic) of numerous microorganisms, such as bacteria, viruses, and fungi. Although most studies have focused on the microbicidal features, there is a lack of reviews on their microbiostatic effects. This review provides a detailed overview of available reports on the microbiostatic activities of ZnO-based nano-materials against different microorganisms. Additionally, the factors that affect the efficacy of these materials, their time course, and a comparison of the available antimicrobials are highlighted in this review. The basic properties of ZnO, challenges of working with microorganisms, and working mechanisms of microbiostatic activities are also examined. This review underscores the importance of further research to better understand ZnO-based nano-materials for controlling microbial growth.

## 1. Introduction

Pathogenic microbes, such as bacteria, viruses, and fungi cause infectious diseases in humans and other living organisms. Bials kill microorganisms and inhibit their growth. They are a broad category of compounds that prevent and treat various infectious diseases in humans, animals, and plants [1,2,3,4,5]. Antimicrobials may also destroy microorganisms that are beneficial to humans. However, other harmful microorganisms can resist the effects of antimicrobial agents and continue to grow. The development of antimicrobial resistance (AMR) in microorganisms is a significant global health concern, as it can render previously effective treatments ineffective and make treating infections more difficult [6,7]. Appropriate and responsible antimicrobial use is crucial to combat AMR, ensure effective treatment, reduce side effects, prevent infections, and maintain human and animal health.

It is crucial to shift strategies in antimicrobial fabrication to respond to AMR challenges, improve efficacy, broaden the spectrum of activities, and explore alternative sources and approaches. This can lead to the development of more effective antimicrobial strategies to combat infectious diseases and promote public health [8,9,10,11]. One of the benefits of continuous advancements in nanotechnology is the introduction of nano-materials with antimicrobial activity [12,13,14,15,16]. Nano-materials containing AgO, ZnO, TiO_2_, SiO_2_, and others have good stability compared to organic materials, which is an important characteristic of antimicrobial materials. Nanoparticles (NPs) and their composites have been shown to have antimicrobial properties [16,17,18,19,20,21,22]. Metal- and metal oxide-based NPs have nonspecific broad-spectrum antimicrobial activities [23,24,25,26,27]. The induction of resistance by microbes is complicated by the non-specific antimicrobial activity of NPs. These NPs are effective microbicidal and microbiostatic agents owing to their unique properties. The antimicrobial properties of polymeric composites and biopolymers have been demonstrated, along with those of modified and unmodified NPs [28]. Blending metals and metal oxides with polymers/biopolymers produces highly improved antimicrobial activities due to their synergistic effects [21,29,30,31,32,33,34,35]. Blending refers to the mixing of metal or metal oxides with polymers or biopolymers to form a uniform mixture. This can be achieved by melting, casting, mechanical mixing, or dissolution, depending on the characteristics of the components.

Various materials are known for their antimicrobial activities, including silver-based [36,37], zinc-based [12,38], and copper-based materials [39,40,41], chitosan [42,43,44], essential oils [45,46,47], nano-materials [16,48,49], enzymes [50,51,52], polyhexanide compounds [53,54,55], clay minerals [56,57], and noble gases [58,59]. Metal-based composites are effective antimicrobial agents. They heal wounds, damage the microbial membranes, and exert long-lasting effects [12,36,39]. Chitosan is natural and useful in medicinal applications. It is safe for the human body, possesses antimicrobial properties, and decomposes in the environment [42,43]. Essential oils, which originate from plants and have a pleasant smell, are helpful against microbes. They can be easily added to different products, although their effectiveness varies significantly [45,46]. Nano-materials have several advantages, including improved surface area and targeted delivery. However, their potential toxicity to the human body and environment remains a major challenge [16,48]. Enzymes, such as endolysin, proteases, and amylase are specific and easily breakable, but are expensive. Polyhexanides, another class of materials, are effective against biofilm formation but may lead to resistance and environmental damage. Clay minerals are abundant, have physical modes of antimicrobial action, and exhibit inconsistencies in antimicrobial performance. Noble gases are inert and non-toxic with a considerable antimicrobial performance. Reports have indicated that further studies should be conducted on their availability, cost, and antimicrobial mechanisms [56,57]. When choosing or fabricating antimicrobial materials for different applications, it is important to consider their effectiveness, safety, and environmental impact. A summary of the categories of these materials based on their active antimicrobial agents is presented in Table 1.

ZnO-based nano-materials are proven materials for various applications, including antimicrobial action [63]. In biomedical applications, ZnO exhibits selective toxicity, biocompatibility, a reactive oxygen species-generating ability, and photochemical stability [64,65,66]. According to the U.S. Food and Drug Administration (21CFR182.8991), ZnO is a safe (GRAS) material. Furthermore, ZnO inhibits the growth of microbes and has been used to preserve and control the spoilage of different types of foods [67]. Mechanisms, such as reactive oxygen species generation, Zn^2+^ release, NP penetration, and membrane deterioration, have been suggested for the antimicrobial actions of ZnO NPs [12,68,69,70,71,72]. These mechanisms can be categorized as chemical or physical. Chemical attacks (microbicidal) kill microorganisms, whereas physical interferences (microbiostatic) inhibit their growth. Although a chemical attack is intended to kill microbes, it can inhibit growth if the concentration of the agent is too low. For healthcare applications, ZnO-based nano-materials must be further evaluated to ensure their safety and efficacy before their widespread use. Furthermore, these nano-materials should be carefully evaluated for their potential toxicity to minimize their adverse effects on humans, animals, and plants. The advantages and drawbacks of these two mechanisms are summarized in Table 2.

Microbiostatic materials or surfaces inhibit the growth of microorganisms without killing them. Microbiostatic materials can be applied to various surfaces and products to prevent the infection and spread of harmful microorganisms. Their numerous uses involve healthcare settings, meal preparation, household products, and other environments where hygiene and infection control are critical [85,86]. The inhibition of bacterial growth is a characteristic of microbiostatic materials [23]. Second, microbiostatic materials have long-lasting effects. A variety of microbiostatic materials are non-toxic to humans; therefore, they are suitable for a range of applications. Microbiostatic materials help prevent the emergence of antimicrobial resistance in addition to targeting specific microorganisms. Although microbiostatic materials can reduce microbial growth, they are not necessarily sterile or capable of eliminating all pathogens. To maintain their normal functions, cleaning and proper hygiene practices are essential, along with the use of microbiostatic materials.

Several factors influence the microbiostatic activity of ZnO-based nano-materials, including size, shape, concentration, stability, exposure time, pH, and surface properties. Increasing the surface area of the NPs and their ability to release Zn^2+^ ions helps smaller particles exhibit higher antimicrobial activity. The surface charge of the ZnO NPs can also affect their interactions with microorganisms. Positively charged NPs have stronger antimicrobial effects, likely because of their enhanced binding to negatively charged microbial cell membranes [87,88,89,90].

This review summarizes the current state of research regarding ZnO-based nano-materials for microbiostatic activity. Additionally, microbiostatic activity evaluation methods, mechanisms of action, and factors affecting the performance of the materials are summarized. Although there are numerous reviews regarding antimicrobial materials, there is no single review that focuses on the selected research area. Additionally, several reviews have been published on ZnO nanostructure-based materials with microbicidal effects, rather than microbiostatic activities. Thus, it is worth updating the progress in ZnO-based nano-materials with microbiostatic activities. In this review, these materials are categorized as unmodified ZnO NPs, modified ZnO NPs, unmodified one-dimensional ZnO nanostructures, and modified one-dimensional ZnO nanostructures. The characteristics of these materials and their microbiostatic performances are discussed in detail in this paper.

An extensive review of scholarly articles was undertaken through academic repositories such as Scopus, Web of Science (WoS), and PubMed to compile this comprehensive summary. The research technique involved the utilization of search terms linked to ZnO nano-materials, their antimicrobial properties, and the microbiostatic impact on various microorganisms. Additionally, backward searching and scrutinizing reference lists also served to highlight pertinent studies. The search was not restricted by a specific time range, allowing for a comprehensive analysis of the available literature up to the present. Research was chosen for its focused examination of the antimicrobial properties of ZnO-based nanocomposites to guarantee alignment with the central theme of this analysis.

## 2. Brief History and Properties of ZnO

Zn is the fourth most used metal worldwide, after Fe, Al, and Cu [91]. Zn is an essential element in the human body and is found in tissues. Zn plays a significant role in the fight against human infections. It has a high reduction ability, thus facilitating the formation of ZnO from Zn. The use of ZnO in nanoscience began in the 1930s [92,93]. Research on ZnO has increased drastically since 1990, although it was developed only in 2000 BC [94,95]. Since its discovery, ZnO has been used in ointments, treatments of different infections, and cosmetic applications owing to its biocompatibility [96]. Although several countries, such as India, Iran, China, Germany, the USA, and France, are associated with Zn/oxide production, large-scale ZnO production methods were introduced in the 19th century by the Americans (direct method) and France (indirect method) [94]. Three ZnO production methods are available: (1) the direct oxidation of Zn metal, (2) ore reduction to metallic Zn with subsequent reoxidation, and (3) oxide precipitation followed by calcination. In the 21st century, ZnO has been extensively studied and established as an inorganic compound with 5 × 10^8^ kg annual production. The applications of ZnO in brass, semiconductors, galvanization, and die casting have been discussed in detail in another review [94]. The synthesis methods for ZnO NPs and their derivatives have been discussed in detail elsewhere [97,98].

One-dimensional ZnO nanostructure and zero-dimensional ZnO nano-materials, such as NPs, are among the most well-known antimicrobials. Both classes of materials are morphologically different and exhibit different properties depending on their chemical modifications [99]. Although nanostructures and ZnO NPs have antimicrobial properties [100,101], they are preferable for such applications [12,102]. However, note that the NPs may also be more cytotoxic to mammalian cells, highlighting the importance of careful consideration when using NPs for biomedical applications [103]. High efficacy, activity against different infectious microorganisms, and cost-effectiveness are among several advantages of using ZnO NPs [17]. Researchers have investigated the antimicrobial activity of ZnO-based materials to exploit these and other aspects, which are discussed below, after a brief introduction to microorganisms and the associated challenges.

## 3. Challenges of Working with Microorganisms

### 3.1. Culturing and Containment Risks

Microbes are ubiquitous and cannot be observed with the naked eye. Microbial culture is a crucial process for in vitro experiments. One of the primary risks is the contamination of cultures with unintended microorganisms. Contamination can occur through airborne particles, improperly sterilized equipment, or cross-contamination between cultures. Contamination can lead to inaccurate experimental results and the unintentional propagation of potentially harmful microorganisms. Certain bacteria, viruses, and fungi cause diseases in humans, animals, and plants. Cultures of pathogenic microorganisms require strict containment measures to prevent accidental exposure or release. Laboratory-acquired infections can occur if proper precautions are not taken, posing a risk to laboratory personnel and the community. Accidental spills or leaks of cultures, contaminated media, or waste can occur during handling, transfer, or disposal. This can lead to environmental contamination or the spread of microorganisms. To reduce the risk of spills and leakages, it is crucial to follow proper containment protocols. These include the use of spill kits, biohazard bags, and appropriate waste disposal methods. Cultivating microorganisms that possess antibiotic-resistance genes can contribute to the proliferation of antibiotic resistance in the environment. If microorganisms that resist antibiotics are set free, or if their genetic material is transferred to other organisms, the effectiveness of antibiotics in medical and veterinary treatments may be impeded. Appropriate containment measures and the responsible use of antibiotics are crucial to mitigate this risk [104,105,106].

To mitigate these risks, laboratories should follow specific biosafety guidelines and regulations, such as the ones provided by the Centers for Disease Control and Prevention in the United States or the World Health Organization [106,107,108]. These guidelines classify microorganisms according to their potential risks and suggest suitable levels of containment and safety measures for each category. Furthermore, it is crucial to ensure the safety of culture and containment practices through the regular training of laboratory personnel, proper waste management, and strict adherence to standard operating procedures.

### 3.2. Development of Resistance

**Figure 1:**
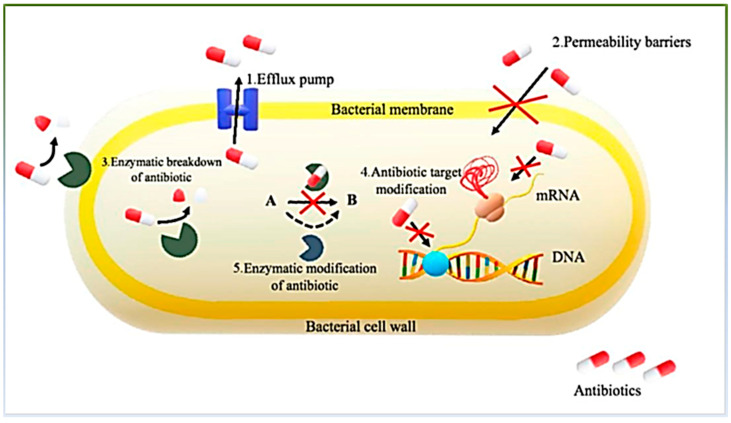
Mechanisms of antimicrobial resistance in molecular level. Reprinted from Ref. [115].

As discussed earlier, the AMR of microbes refers to the microbe’s ability to resist the effect of the antimicrobial agent to which it was designed to eliminate them and is one of the most challenging issues in public health and sustainable economic development. A highly influential report estimated that approximately 10 million people could perish annually by 2050 because of AMR [68,82,109,110,111]. In addition to being a major global health problem, AMR has become a challenging problem for researchers designing and synthesizing drugs for specific microbes that are not vulnerable to resistance. AMR can arise from natural processes (intrinsic), genetic mutations (acquired), inaccurate diagnoses, and the overuse or misuse of drugs. As depicted in Figure 1, the molecular mechanisms of AMR include the inactivation of antibiotics, target protection, target site modification, active efflux, target bypass, decreased influx, and downregulation [112,113,114,115]. These factors have been discussed in detail [116,117]. Inconsistencies in duplicating the performance of the materials include the results of antimicrobial resistance and non-standardized testing methods of antimicrobial activity (agar dilution and disk diffusion) [118].

Integrated R&D programs are necessary to overcome AMR, and these efforts require highly trained experts and monetary investments. Nano-materials, especially NPs, can overcome the capacity of microbes to develop resistance owing to their unique characteristics [14]. Creating societal awareness regarding the proper use of drugs could minimize this overall problem. Most antimicrobials, either for humans or animals, are inexpensive and available in healthcare centers. Thus, enhancing awareness of the proper use, handling, and management of waste is crucial. While antimicrobial activity tests are performed on a laboratory scale (as observed in most reports), the low concentration of microbes tested for large amounts of antimicrobial agents is not representative of the actual situation. Similarly, in vitro experiments may provide reproducible performances, but the reality test (in human or animal bodies) may not have a similar effect. Such tests would highlight only the potential for the intended purposes.

## 4. Antimicrobial Activity of ZnO

### 4.1. Time Course of ZnO for Antimicrobial Activity

Durable antimicrobials provide long-term defense against germs. They work well in numerous applications because they do not require repeated applications. A US Environmental Protection Agency (EPA) report showed that the EPA endorsed a long-lasting antimicrobial agent in 2020 [119]. As a part of this continuous effort, researchers are exploring long-lasting antimicrobial materials for public use. Among these, ZnO-based materials have emerged as promising candidates owing to their distinctive physicochemical characteristics and broad-spectrum efficacy. The time course of the antimicrobial action of ZnO-based materials is a compelling process affected by different factors, such as the constituents of the composite, concentration, and microbial target. According to the available reports, ZnO-based materials can be effective for minutes [12,120], hours [12,121], days [122,123,124], weeks [125,126], and months [126]. The most long-lasting antimicrobials are synthesized through the methods of ZnO NPs incorporation, encapsulation, surface coating, and slow-release formulations [124,125,126]. These approaches involve the incorporation of antimicrobial agents into materials or coatings to provide sustained protection for various applications [127]. An overview of the time course of the antimicrobial activity of ZnO-based materials is provided in Table 3.

### 4.2. ZnO NP-Based Materials

**Figure 2:**
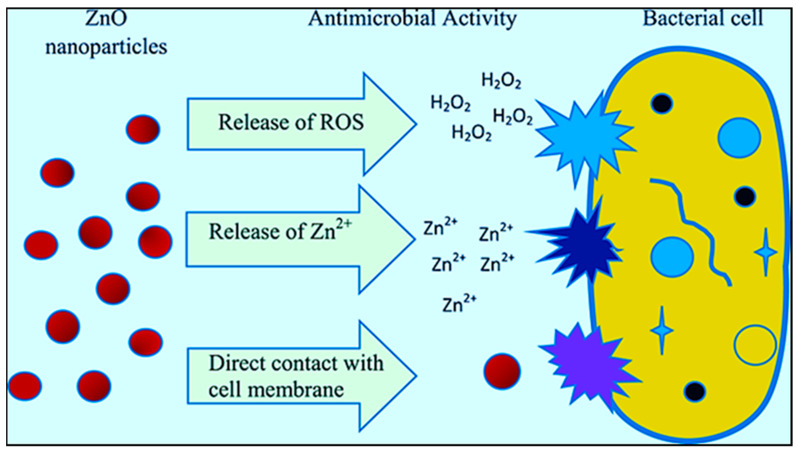
Antimicrobial activity mechanism of ZnO nanostructures. Reprinted from Ref. [129].

Gram-negative and Gram-positive bacteria are classified according to their cell wall type. Both categories contain a peptidoglycan layer and a cytoplasmic membrane. Gram-negative bacteria also contain lipopolysaccharides in their outer membranes, whereas Gram-positive bacteria contain polymeric technoic acids [14]. Both bacterial membranes have negative charges due to carboxylate- and phosphate-containing groups (Gram-negative and Gram-positive, respectively) [72]. The negative charges on the membrane serve as binding sites for metals and other environmental conditions. NPs’ antimicrobial action is through reactive oxygen species generation, disruption, penetration, and interactions with proteins and DNA [128]. Figure 2 shows the mechanisms underlying the antimicrobial activities of ZnO nanostructures.

The antimicrobial activity of ZnO NPs can be enhanced by modifying their surface properties, such as by coating them with polymers, peptides, or other biomolecules, which can improve their stability and selectivity toward target microorganisms. ZnO NPs exhibit relatively low toxicity in mammalian cells, making them promising candidates for developing novel antimicrobial agents for various biomedical applications, including wound healing, dental materials, and medical implants [12,130,131]. As shown in Table 4, unmodified ZnO NPs show considerable potential as microbiostatic agents and can be used in a variety of applications to prevent the growth of microorganisms.

**Figure 3:**
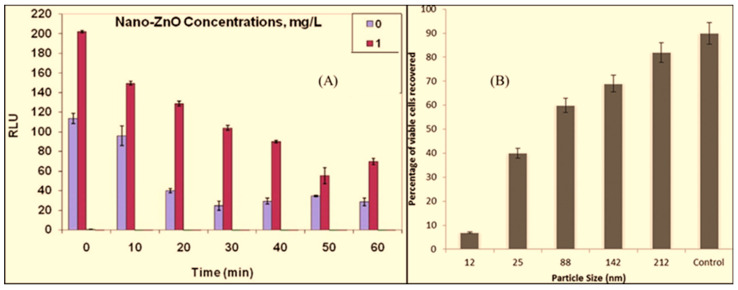
(**A**) Response of *P. putida* microbe to nano-ZnO. Reprinted from Ref. [133]. (**B**) Effect of size of ZnO NPs on growth of methicillin-sensitive *S. aureus* strain. Reprinted with permission from Ref. [135]. Copyright 2011 American Chemical Society.

Researchers have used unmodified ZnO NPs for microbiostatic activities against different microorganisms, such as *Streptococcus mutans* (*S. mutans*) [132], *Pseudomonas putida* (*P. putida*) [133], *Streptococcus pyogenes* (*S. pyogenes*) [134], *Staphylococcus epidermidis* (*S. epidermidis*), *Enterococcus faecalis* (*E. faecalis*), *Bacillus subtilis* (*B. subtilis*), *Escherichia coli* (*E. coli*), *Proteus vulgaris* (*P. vulgaris*), *Salmonella typhimurium* (*S. typhimurium*), *Shigella flexinari* (*S. flexinari*), *Pseudomonas alcaligenes* (*P. alcaligenes*), *Enterobacter aerogenes* (*E. aerogenes*) [135], *Mycobacterium tuberculosis* (*M. tuberculosis*) [136], *Staphylococcus aureus* (*S. aureus*), *Pseudomonas aeruginosa* (*P. aeruginosa*), *Serratia marcescens* (*S. marcescens*), and *Klebsiella pneumonia* (*K. pneumoniae*) [137]. These ZnO NPs were either purchased from the market or synthesized using different methods (refer to Table 4). The common findings of these reports are as follows: (1) bulk ZnO is less toxic (toward microbes) than ZnO NPs and (2) the toxicity of ZnO NPs depends on the size of the NPs, visible light, and dose of the NPs. Note that particle size and light play significant roles in microbiostatic activity, especially for ZnO NPs, compared to other metal–metal oxide NPs [27]. The abscissa and ordinate in Figure 3A show the time (min) and changes in lux output (relative light units, RLUs), respectively, for a *P. putida* strain exposed to 0 and 1 mg/L ZnO NPs. From 0 to 60 min, there was a light output loss from the biosensor, indicating the toxicity of the ZnO NPs toward the strain. Similarly, as the size of the ZnO NPs was increased from 12 to 212 nm, the number of viable cells recovered increased from approximately 7% to 82% (Figure 3B), showing that the smallest size (12 nm) inhibited the growth of the *S. aureus* strain by approximately 97%. According to these reports, the bacteriostatic activity of ZnO NPs was achieved through the release of Zn^2+^ ions and generation of reactive O_2_ species (ROS). In addition, some microbes, such as *E. coli*, may lose their integrity after exposure to ZnO NPs [138].

**Table 4:** Microbiostatic activity and other characteristics of bare ZnO NPs.

Material	Target Microbe	Time, Temp	Synthesis Method	Note	Ref.
ZnO NPs	*S. mutans*	24 h, 37 °C	Precipitation–diffusion	The inhibition ability was determined using the liquid dilution method. The minimum inhibition concentration (MIC) was found to be 500 ± 306.18 μg/mL. Size reduction in the NPs increases the contact surface and improves the performance. NP size = 125 nm	[132]
	*E. coli*, *S. aureus* *P. aeruginosa*, *E. faecalis*, *P. aeruginosa*, *A. baumannii*	24 h, 37 °C	Plant-mediated biosynthesis	Aristolochia indica leaf was served as a source for the NP synthesis. The MIC was determined via Macro-broth dilution. With a size of 22.5 nm and zeta potential of −21.9 ± 1 mV exhibited, the MIC increased from 25 to 200 μg/mL. NP size = 50–70 nm.	[139]
	*Pseudomonas putida* KT2440	24 h, 28 °C	N/A	Commercial ZnO NPs were used. Assayed via dilution plating on salt-free Luria Broth. Bulk equivalents of these NPs showed no inhibitory activity, indicating that particle size was determinant in activity. Zn ions and nano-ZnO were effective bacteriostatic agents, unlike the bulk-ZnO in 10 mg Zn/L. NP size < 100 nm.	[133]
	*S. pyogenes*	24 h, 37 °C	Commercial product	Shape: Spherical with rod mixture. The turbidity method was used to determine the bacteriostatic effect of ZnO NPs. The turbidity of the bacterial suspension treated with 10, 50, and 100 μg/mL of ZnO was reduced by 35.75 ± 5.28, 70.29 ± 6.86, and 81.18 ± 5.70%, respectively, within 24 h. Binding ZnO to bacterial cell wall: Electrostatic force between Zn^+^ and anionic groups on bacterial cell wall. NP size = 31.4–66.3 nm.	[134]
	*S. epidermidis, S. pyogenes*, *E. faecalis*, *B. cereus*, *P. vulgaris*, *S. typhimurium S. flexinari*, *P. alcaligenes*, *E. aerogenes*		Room temperature and solvothermal	Methicillin resistant and sensitive strains were tested. A 4–7 mM colloidal suspension of ZnO NPs inhibited > 95% of growth for most of the microorganisms, except *S. typhimurium*, as its growth was inhibited by 50% under ambient lighting conditions. The release of free Zn^2+^ ions from ZnO had minimal effect on the performance. Bacteriostatic activity of ZnO NPs: through the accumulation of NPs in the cytoplasm or on the outer membranes. NP size = 12–307 nm.	[135]
	*M. tuberculosis*	24 h, 37 °C	Chemical precipitation	A Microplate Alamar Blue Assay (MABA) was used to determine the MIC of ZnO; 1 μg/mL of ZnO was the lowest concentration inhibiting the growth of the bacteria. ZnO NPs did not show bactericidal effect against *M. tuberculosis*. NP size = 9.3 ± 3.9 nm.	[136]
	*S. epidermidis*, *S. pyogenes*, *S. marcescens*, *K. pneumoniae*, *P. aeruginosa*	24 h, 37 °C	Facile microplasma	Shape: Nanosheets (40–50 nm size), nanodrums, and nanoneedles. ZnO used: 1 mg/mL. The antibacterial activity of the ZnO nanostructures was determined using the Agar well diffusion method. A maximum inhibition zone of 21 mm was recorded for *S. marcescens*. Growth inhibition was higher in ZnO dissolved in dimethyl sulfoxide than that of dry ZnO powder. Mechanism: release of Zn^2+^ ions and a higher surface area-to-volume ratio.	[137]

As discussed above (Table 4), ZnO NPs have gained significant attention in recent years owing to their microbiostatic activities. Although they offer several advantages, they also have certain drawbacks that need to be considered. ZnO NPs can agglomerate and lose their desired properties, such as surface area and reactivity, owing to their inherent tendency to form aggregates. Aggregation can affect stability, dispersion, and overall antimicrobial efficacy. Another problem is their nonspecific activity; ZnO NPs exhibit broad-spectrum antimicrobial activity, which implies that they can target a wide range of harmful and beneficial microorganisms. Thus, to minimize the associated concerns and gain additional uses, the modification of ZnO NPs is vital.

**Figure 4:**
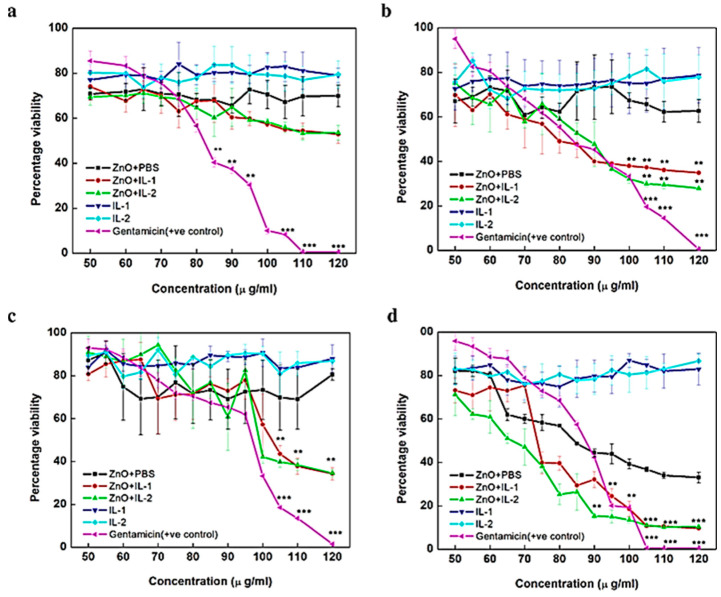
Growth inhibition studies of ZnO+PBS, ZnO+IL1, ZnO+IL2, IL1, IL2, and gentamicin at the concentration range of 50−120 μg/mL in (**a**) *E. coli*, (**b**) *B. subtilis*, (**c**) *K. pneumoniae*, and (**d**) *S. epidermidis*. PBS-treated cells were treated as a negative control. Error bars represent standard error with respect to the mean of three biological replicates. ** *p* < 0.001, *** *p* < 0.0001. Reprinted with permission from Ref. [147]. Copyright 2018 American Chemical Society.

Chemically modified ZnO NPs show promise for microbiostatic applications. The antibacterial properties of the NPs were enhanced through the the addition of several chemical groups. Some of the most commonly used modifications include coating ZnO NPs with metals/oxides [23,140,141,142,143], organic compounds, and polymers [137,144,145,146,147,148] (Table 5). Some advantages of polymeric NP composites for antimicrobial activity [149,150,151,152] are as follows. (1) Controlled release: Polymeric NP composites can be designed to have a controlled release of antimicrobial agents. A sustained antimicrobial effect can be maintained by releasing antimicrobial agents at a controlled rate over time. (2) Targeted delivery: polymeric NP composites can target specific microorganisms. Therefore, this can increase antimicrobial effectiveness and reduce side effects by delivering the agents directly to the site of infection. (3) Improved stability: Polymeric NP composites can improve the stability of antimicrobial agents by reducing their degradation. This can increase their shelf life and ensure that they are effective for longer periods. Polymeric NP composites reduce the toxicity of antimicrobial agents. Polymeric NP limit the interaction of agents with healthy cells and tissues, thereby reducing the risk of toxicity. (4) Enhanced penetration: Polymeric NP composites enhance the penetration of antimicrobial agents into biofilms and other difficult-to-reach regions. This can improve the effectiveness of the agents and reduce the risk of resistance. A range of advantages make polymeric NP composites effective antimicrobial agents. In addition, modifiers have been used to solve the ZnO NP aggregation problem during antimicrobial tests [153,154]. Furthermore, ZnO NPs have been enhanced with an ionic liquid by acting as both an antimicrobial agent and a dispersion medium (Figure 4) [147]. In this study, two ionic liquids were used to disperse ZnO NPs, namely, choline acetate and 1-butyl-3-methylimidazolium chloride, abbreviated as IL1 and IL2, respectively, and tested against *E. coli*, *B. subtilis*, *K. pneumoniae*, and *S. epidermidis*. The highest efficiency was obtained when the ZnO NPs were dispersed in IL1 and IL2 (ZnO + IL1 and ZnO + IL2) and tested against *S. epidermidis* (Figure 4D). The ZnO NPs were dispersed in phosphate-buffered saline (PBS) for comparison.

**Figure 5:**
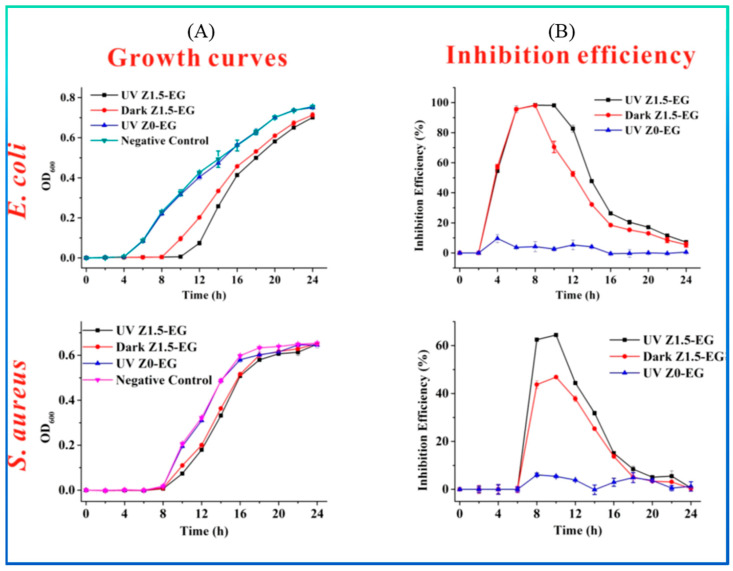
Growth curve (**A**) and inhibition efficiency (**B**) of *E. coli* and *S. aureus* exposed to the ethylcellulose/gelatin fibers with and without UV light. Reprinted with permission from Ref. [144]. Copyright 2018 American Chemical Society.

Liu et al. reported the role of UV irradiation in enhancing the bacteriostatic activity of ZnO NPs supported by ethylcellulose/gelatin (EG) fibers against *E. coli* and *S. aureus* [144]. As shown in Figure 5A, EG fibers containing 1.5 wt. % ZnO NPs (Z1.5-EG) have shown better antimicrobial activity with UV exposure (UV Z1.5-EG) than UV-protected (Dark Z1.5-EG) materials against the target strains. The EG fiber without ZnO NPs but with UV exposure (UV Z0-EG) was also tested against the selected microbes and showed almost no antimicrobial activity (Figure 5B). OD_600_ (shown in the ordinate of Figure 5A) is the optical density at 600 nm, where the bacterial culture was measured [155].

**Figure 6:**
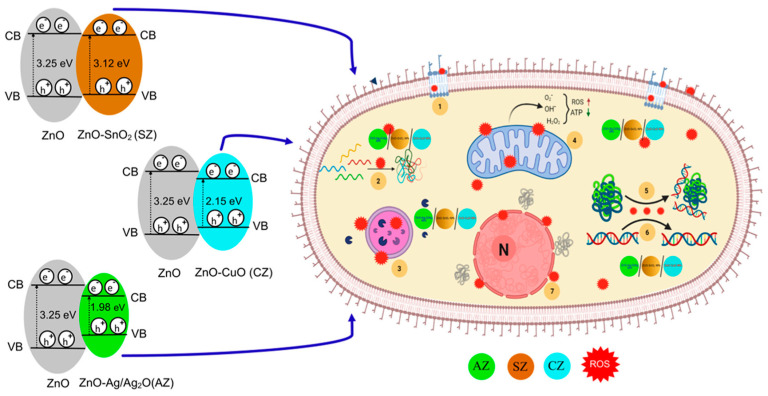
Change in the band energy gap for metal/oxide-modified ZnO NPs and their antimicrobial mechanisms. Reprinted from Ref. [143].

In another study, mixtures of metal oxides and ZnO NPs were prepared and their synergistic effects on antimicrobial activity were investigated [143]. The antimicrobial activities of the materials (ZnO−CuO, ZnO−Ag_2_O/Ag, and ZnO−SnO_2_ NPs, prepared using the in situ reduction method) increased as their concentration increased. Of these mixed metal NPs, ZnO−AgO_2_/Ag showed the best performance even at low concentrations (50 μg/mL) in 3 h against *P. aeruginosa*, *A. baumannii*, *K. pneumoniae*, and *C. albicans*. In comparing the band energy gaps (Eg) of the bimetallic oxide NPs, ZnO−AgO_2_/Ag produced the lowest value (1.98 eV) (Figure 6), and the authors hinted that there might be a relationship between the Eg and antimicrobial performance, although clear evidence is lacking. Another report [156] indicated that a reduction in Eg increased the photocatalytic activity of NPs. This might enhance the generation of ROC, subsequently increasing the antimicrobial activity. Generally, bimetallic mixed-oxide NPs show better antimicrobial efficiency than single-metal oxide NPs. Other ZnO NP-based materials are listed in Table 5, including the type of material, target microorganism, synthesis method, particle size, and other characteristics. The detailed synthesis mechanisms of ZnO NPs and their composites are discussed in another review [157].

**Table 5:** Microbiostatic activity and other characteristics of modified ZnO NPs.

Material	Target Microbe	Time, Temp	Synthesis Method	Note	Ref.
ZnO-EG ^a^	*E. coli* and *S. aureus*	24 h, 37 °C	Electro-spinning	Antimicrobial activity was performed using the disc diffusion method; 1, 1.5, and 2 wt.% ZnO NPs showed inhibitory diameters of 0.69, 1.30, and 1.61 mm/mg against *E. coli* and 0.75, 1.17, and 1.33 mm/mg against *S. aureus*, respectively. Efficiency was enhanced via UV irradiation. Excellent hydrophobicity, water stability, and antibacterial performance. NP size = 30 nm.	[144]
ZnO-GPTMS ^b^	*E. coli* and *S. aureus*	24 h, 37 °C	Sol−gel method and surface modification	The preparation of the bacterial inoculum was carried out using the McFarland scale. The reaction time of the ZnO NP synthesis did not make changes in size or antibacterial activity. Antibacterial results with different treatments were better for *S. aureus* compared to *E. coli*. Parameters such as dyeing, softening, and number of washes did not affect the efficiency. NP size = 5 nm.	[145]
ZnO-L-RMGIC ^c^	*Cariogenic*	24 h, 37 °C	Probe sonication	NP size ranged from 10 to 150 nm. Zinc ion was released from the NPs. The highest Zn ion releases over 1, 14, and 28 days were 12.59, 13.5, and 14.1 mg/L, respectively. After 24 h, the highest and the lowest bacterial count were 2.79 × 10^4^ and 1.5 × 10^3^ cfu/mL, respectively.	[146]
ZnO-ILs ^d^	*E. coli*, *B. subtilis*, *K. pneumoniae,* and *S. epidermidis*	24 h, 37 °C	Precipitation and dispersion	ZnO NPs (60 nm) were dispersed in choline acetate and 1-butyl-3-methylimidazolium chloride to avoid aggregation. The ionic liquids served for dispersion and as an antibacterial agent. ZnO NPs exhibited the highest antibacterial activity in 1-butyl-3-methylimidazolium against *S. epidermidis*. The production of ROS increases efficiency.	[147]
ZnO-NFC ^e^	*S. aureus*, *B. cereus*, and *K. pneumoniae*	20/24 h, 30/37 °C	Electrostatic assembly	The AATCC 100 standard test method was used to assess the antimicrobials activity. In total, 4 mg of the composite suspension (100 μL of nutrient broth) or 1.5 cm by 1.5 cm specimens of a coated paper sheet (100 μL of a solution of 12.5% diluted nutrient broth) were used. The test was performed in the presence and absence of light. NP size = 40.7 ± 14.5 nm.	[158]
ZnO-PVP/PVA/PGA ^f^	*E. coli* and *S. aureus*	24 h, 37 °C	Hydrothermal	ZnO NPs were stabilized using PVP, PVA and PGA polymers; 2.1 × 10^7^ CFU/mL and 4.1 × 10^7^ CFU/mL of *S. aureus* and *E. coli*, respectively, were used. Cell reduction activity of ZnO NP was performed using the colony count method in liquid. NP size = 30–100 nm.	[159]
ZnO-PDDA/RMGM ^g^	*E. coli*	48 h, 37 °C	Hydrothermal	The standard plate counting method was used for the antimicrobial effect. About 107 CFU/mL of *E. coli* was used. It is reusable with a rate of over 98%. NP size = 16.95 nm.	[160]
ZnO, ZnO-PVA ^h^	*E. coli* and *S. aureus*	24 h, 37 °C	Solvothermal	Antimicrobial activity was analyzed using an agarose diffusion assay. The density of bacterial cells in the liquid cultures was measured at a 600 nm wavelength. Cell suspension for antibacterial activity was 1 × 10^5^ colony-forming units (CFUs) mL^−1^. The MIC was determined using a modified resazurin method. In total, 100 μL of nutrient broth or sterile saline was used on the plates, and a 5 × 10^6^ CFU/mL bacterial suspension was added. ZnO-PVA was used for anti-infection (female mice, 5 × 10^6^ CFU/mL *E. coli* in 50 μL of sterile phosphate-buffered saline). NP size = 4 nm.	[161]
ZnO/SBA ^i^	*E. coli* and *S. aureus*	24–72 h, 37 °C	Co-condensation/impregnation/calcination	In total, 2 mg of ZnO/SBA powder was added to 20 mL of LB agar, and 100 μL of each of *S. aureus* (10^5^ CFU mL^−1^) and *E. coli* (10^5^ CFU mL^−1^) were used. Photocatalytic antibacterial activity. SBA/ZnO showed a bacteriostatic effect with inhibition rates of 32.61 and 38.33% against *E. coli* and *S. aureus*, respectively. NP size = 40 nm	[162]
ZnO/Ag-Haw ^j^	*E. coli* and *S. aureus*	24 h, 37 °C	Template-oriented precipitation/sol–gel method	ZnO/Ag-HAw was sintered at 600 C for 10 h before use. ZnO/Ag-HAw showed non-cytotoxicity, and ZnO had an average particle size less than 30 nm. Monkey bone marrow mesenchymal stem cells were used. Antimicrobial activity was investigated using the plate colony-counting method. The measured ZnO in the sample was 9.97 wt.%, which was about 66.5% of the theoretical value. The material had a better antibacterial effect against *S. aureus* than *E. coli*.	[140]
ZnO-PLGA ^k^	*E. coli*	24 h, 37 °C	Laser ablation/low-temperature technology	Rod-like ZnO with an average hydrodynamic NP diameter of 47 nm (90% ZnO and 10% metallic Zn). The number of cells on surface of the composite with 0.001% and 0.01% ZnO decreased by 2 and 10 times, respectively. The PLGA–ZnO NP composite containing 0.1% ZnO NPs had bacteriostatic properties. At ZnO NP concentrations of 0.001%, 0.01%, and 0.1%, the rate of 8-oxoguanine formation in DNA increased 1.5, 2.3, and 2.8 times, respectively. PLGA had no antibacterial effect.	[163]
ZnO/PVA/Cel ^l^	*C. albicans*, *E. coli*, and *S. aureus*	24 h, 30/37 °C	Molding	An antibacterial test was performed using the viable shake-flask method. Colony: 10^5^–10^6^ CFU/mL. Solution shaken at 150 rpm at a certain temperature (bacteria: 37 °C, fungus: 30 °C) for 24 h in a water bath oscillator. The thickness of the film was 63–69 μm. Zn^2+^ reached a maximum release value of 4.20 mgL^−1^ after 24 h.	[164]
ZnO-PHB ^m^	*E. coli* and *S. aureus*	24 h, 37 °C	Electro-spinning and electrospraying	It has an average porosity of around 85% and is thermally stable, and 3 and 5 wt.% ZnO were used to form the composite. The growth inhibition by ZnO-PHB was about 95–97%. The PHB alone did not inhibit bacterial growth. NP size = 8–20 nm	[165]
ZnO-PLA-SiO_2_ ^n^	*S. aureus*	18 h, 37 °C	sol–gel method and coating	When 1.5% ZnO and 1.5% SiO_2_ were used, the highest growth inhibition was 20%. SiO_2_ reduced the bacterial inhibition capacity. With an increase in ZnO and SiO_2_ contents, the bacteriostatic effect was disturbed. Only PLA + 1% ZnO was effective bactericidal (90% bacterial cell growth inhibited); 1% ZnO + 1% SiO_2_—bacteriostatic property.	[166]
ZnO/PAN@NFMs ^o^	*E. coli* and *S. aureus*	24 h, 37 °C	Solution blow-spinning	Antimicrobial activity was evaluated using a plate count method assay. For *S. aureus*, the bacteriostatic rate can reach 100%. For *E. coli*, the best antibacterial effect was achieved when the mass of ZnO NPs was 5 wt.%, and the bacteriostatic rate can reach 99.9%. The bacteriostatic rate for E. coli remained 99% after 10 cycles. NP size = 32.8–40.7 nm	[167]
ZnO/TiO_2_ ^p^	*E. coli* and *S. aureus*	24 h, 37 °C	Hydrothermal	Size: 100 nm ≥ diameter of the particles, and the composite displayed a rhomboid shape. Synthesis temperature affects the performance. The maximum bacteriostatic activity reached 99 and 90% against *S. aureus* and *E. coli*, respectively. Antibacterial mechanism: through the ROS formation and release of Zn^2+^ ions. The smaller the size of the ZnO/TiO_2_ nanoarray, the stronger the piezoelectric and antibacterial activity.	[23]
ZnO-SCF/PEEK ^q^	*E. coli* and *S. aureus*	24 h, 37 °C	In situ/hydrothermal	The addition of ZnO improves the binding force between the SCF and PEEK. The composite has good wear resistance too. The composition of ZnO, SCF, and PEEK with 7.5, 15, and 77.5 wt.%, respectively, has the best antimicrobial effect. It produced diameters of 28.9 and 22.2 mm for *E. coli* and *S. aureus*, respectively.	[148]
Sb-ZnO Mg-ZnO	*E. coli*, *S. aureus*, *Saccharomyces*, and *A. niger*	18/24 h, 37 °C	Sol–gel method	The bacteriostatic rate of Sb-doped ZnO was only 12% as the plates were incubated in the dark. Under irradiated incubation, Mg-ZnO showed an improvement in its bacteriostatic rate from 9.8% without irradiating to 83.5%. However, the bactericidal effect was higher than the bacteriostatic effect.	[142]
CTS/-ZnO ^r^	*E. coli* and *S. aureus*	24 h, 37 °C	Room temp. and casting	A nano-ZnO solution was prepared with particle sizes of 5 μm, 100 nm, and 50 nm. The smaller the particle size of the ZnO, the greater the bacteriostatic activity observed. The composite material had a better inhibitory effect on *S. aureus* than on *E. coli*. The material containing 0.3% of 50 nm nano-ZnO had the best antibacterial effect on both target microbes.	[168]
CA/ZnO/Ag NPs ^s^	*E. coli* and *S. aureus*	24/108 h, 37 °C	Electro-spinning	Antibacterial activity was evaluated using the Kirby Bauer disc diffusion assay, performed on an agar plate and in liquid medium. The material effectively inhibited the growth of both the strains up to 108 h; 100% bactericidal effect (0% viable cells) against both strains. NP size = 17.85 nm.	[141]
ZnO-carvacrol	*C. jejuni*	48 h, 37 °C	N/A	ZnO NPs and carvacrol were tested separately and combined. ZnO NPs: <12.5 μg/mL had little inhibition effect, and bacteriostatic and bactericidal effects with 25 and 50 μg/mL, respectively. Synergistic: carvacrol had a better effect than ZnO NPs. ZnO NP effect: physically induce cell leakage.	[169]
ZnO-Mk ^t^	*S. aureus*, *L. fusiformis*, *P. vulgaris,* and *Pr. vermicola*	24 h, 37 °C	Co-precipitation	The microbiostatic effect of Mk-ZnO NPs was determined through the MIC, live and dead, and antibiofilm assay. Mk-ZnO NPs inhibit the growth of Gram-positive and Gram-negative bacteria at 40 and 50 μg/mL, respectively. A 90–50% cell viability at concentrations of 10–100 μg/mL. It also exhibited a mosquito larva controlling capacity. NP size = 10–15 nm.	[170]
ZnO@PVA/KGM ^u^	*E. coli* and *B. subtilis*	24 h 37 °C	Electro-spinning and ultra-sonication	The material was treated @140 °C in citric acid to improve water insolubility. The highest antibacterial activities for *E. coli* and *B. subtilis* were found in 1.0 and 2.0 wt.% ZnO@PVA/KGM, respectively. When the ZnO content is >1.0 wt.%, the antibacterial activity for *E. coli* decreased. Reason: as the value of ZnO NPs increased, the particles gathered into clusters randomly. The material has good photocatalytic activity and filtration efficiency. NP size = 30 ± 10 nm.	[171]
ZnO-ALG ^v^	*E. coli*	48 h, 37 °C	Electro-spinning	Thin and homogeneous nanofiber with a size of 100 ± 30 nm. It exhibited good stability for more than 10 days in physiological conditions. It has similar mechanical properties as human skin. It has 21.0 ± 3.5 MPa and 6.0 ± 1.3% in tensile strength and elongation break, respectively.	[172]
ZnO-MO ^w^	*P. aeruginosa*, *A. baumannii*, *K. pneumoniae*, and *C. albicans*	3–24 h, 37 °C	Solvo-chemical/reduction	ZnO−Ag_2_O/Ag, ZnO−CuO, and ZnO−SnO_2_ composite NPs (<4 nm) were synthesized to gain broad-spectrum activity. The broth dilution method showed the MIC for A. baumannii as the best result. The antibacterial activities of the samples were investigated using the Luria broth (LB) method. Highly effective antibacterial activity was obtained at 12 h of incubation, and the ZnO−AgO_2_/Ag composite was the best. ZnO−AgO_2_/Ag showed high antibacterial activity after just 3 h at a 50 μg/mL.	[143]

^a^ ZnO-ethylcellulose/gelatin, ^b^ ZnO-(3-glycidyloxypropyl)trimethoxysilane, ^c^ ZnO-lignin-resin-modified glass ionomer cement, ^d^ ZnO-ionic liquids, ^e^ ZnO-nanofibrillated cellulose, ^f^ ZnO-polyvinyl pyrrol-idone/polyvinyl alcohol/and poly (L-glutamic acid), ^g^ ZnO-cation polyelectrolyte diallyl dimethylammonium/chloride Red Mud Granular Material, ^h^ ZnO-poly(vinyl alcohol), ^i^ ZnO-Santa Barbara Amorphous, ^j^ ZnO-Ag-hydroxyapatite whiskers, ^k^ ZnO-poly (lactic-co-glycolic acid), ^l^ ZnO-Polyvinyl alcohol/Cellulose, ^m^ ZnO-poly(3-hydroxybutyrate), ^n^ ZnO-polylactic acid/silica, ^o^ ZnO/polyacrylonitrile hybrid nanofiber mats, ^p^ ZnO-titanium dioxide, ^q^ ZnO-surface of acidified short carbon fiber/Poly(ether ketone), ^r^ chitosan-ZnO, ^s^ Cellulose acetate/ZnO/Ag NPs, ^t^ ZnO-Murraya koenigii berry, ^u^ ZnO@poly(vinyl alcohol) (PVA) and konjac glucomannan, ^v^ ZnO-Alginate, ^w^ ZnO-metal oxides.

### 4.3. One-Dimensional ZnO Nanostructures and Their Composites

Although ZnO NPs have a higher surface area and potentially enhanced antimicrobial properties, the one-dimensional ZnO nanostructure demonstrates significant antimicrobial activity. The particle size, surface area, and zinc ion release rate can affect the effectiveness of one-dimensional ZnO nanostructures. However, note that studies primarily focus on ZnO NPs due to their unique properties and potential applications. When one-dimensional ZnO nanostructures come into contact with an appropriate medium, they release Zn^2+^ ions, thereby exhibiting antimicrobial activity. This property has been utilized in various applications, where the antimicrobial activity of ZnO helps prevent the growth of harmful microorganisms, which is a microbiostatic effect (Table 6).

**Figure 7:**
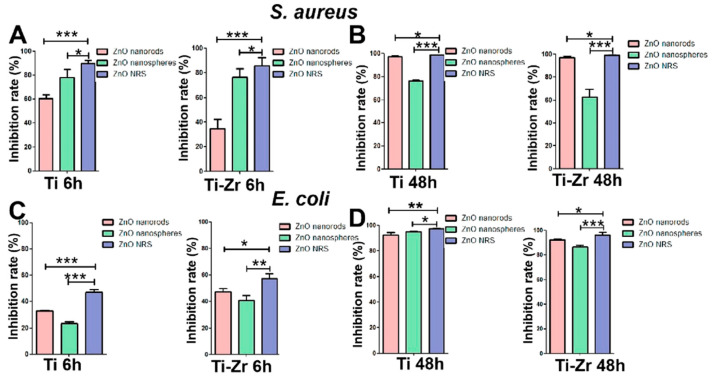
Antibacterial effect of different ZnO samples against *E. coli* and *S. aureus* and under different incubation times in vitro. ZnO samples co-cultured with bacteria for 6 h (**A**,**C**) and 24 h (**B**,**D**). * *p* > 0.05, ** 0.01 < *p* < 0.05, and *** 0.001 < *p* < 0.0. Reprinted with permission from Ref. [175]. Copyright 2020 American Chemical Society.

Accordingly, one-dimensional ZnO nanostructures with different shapes, such as polypropylene-modified ZnO nanowires (ZnO NW-PP), ZnO nanorods (ZnO NR), ZnO rods, ZnO plates, and ZnO nanospheres, have been synthesized using different methods and tested for microbiostatic activity against selected microorganisms (refer to Table 6) [173,174,175,176]. Wang et al. prepared ZnO nanospheres and ZnO nanorods and loaded them onto the surfaces of titanium and titanium–zirconium (Ti-Zr) implants to enhance their antimicrobial activities against *S. aureus* and *E. coli* [175]. As indicated in Figure 7A–D, the materials showed >95% antimicrobial activity after 48 h against both microbes. ZnO nanospheres and ZnO nanorods were also separately loaded onto the surfaces of the implants and showed less antimicrobial activity after 6 h of their composites. However, the ZnO nanorods exhibited almost equivalent effects as the composite material after 48 h. The ZnO nanorods exhibited better long-term antibacterial activity than the ZnO nanospheres owing to the slow release of their larger particles (compared to the ZnO nanospheres). However, the nanospheres were released faster because of their smaller particle size and showed better short-term antimicrobial activity against the target microbes.

Generally, ZnO-based nano-materials with elongated shapes, such as nanorods or nanowires, appear to be more effective in penetrating and disrupting microbial cells, leading to improved microbiostatic activity against target microbes. In addition, the shape of the ZnO-based nano-materials can influence their physicochemical interactions with microbial cells. Moreover, ZnO-based nano-materials with rough or porous surfaces tend to have larger surface areas than those with smoother surfaces, leading to improved efficacy.

## 5. Conclusions and Outlook

Nano-materials modified with ZnO have recently attracted significant attention because of their potential antimicrobial properties. Several studies have demonstrated the microbiostatic activity of these materials against bacteria, fungi, and viruses. The broad-spectrum activity of this microbial compound is important because it targets a wide variety of pathogens and reduces the risk of microbial resistance. Numerous studies have shown that ZnO NPs exhibit a strong microbiostatic activity. ZnO releases zinc ions (Zn^2+^) that penetrate microbial cells and disrupt their structure and function. In addition to releasing zinc ions, light and moisture can generate reactive oxygen species from ZnO NPs. It is possible to cause oxidative stress and damage microbial cell components with ROS, such as superoxide and hydroxyl radicals. Researchers have explored various strategies for enhancing the Zn^2+^ release and maintaining the microbiostatic activity of ZnO. The surface modification of nano-materials with ZnO NPs or other organic materials enhances the dispersibility of the particles and stability of the overall structure. Zn^2+^ can also be sustained using controlled-release systems, ensuring prolonged antimicrobial efficacy. Among other applications, biomedical devices, wound healing, and food packaging benefit from nano-materials’ antimicrobial effects.

For practical applications, several challenges must be overcome despite the nano-materials’ promising microbiostatic properties. Standardized testing protocols are required to address concerns regarding cytotoxicity and potential environmental impacts. ZnO-based nano-materials should be investigated for improved biocompatibility and safety profiles, as well as better microbiostatic efficacy evaluation procedures. Additionally, these materials have long-lasting antimicrobial potential for public use; however, further investigation is required.

Nano-materials based on ZnO have demonstrated significant potential in microbiostatic applications. Their strong antimicrobial activity, broad-spectrum activity, and tunable properties make them attractive candidates for healthcare and food safety applications. However, research and development are required to address these challenges and ensure the safety and effectiveness of practical applications. Additionally, to enhance the microbiostatic activity, it is important to consider the impact of shape during the design of the materials. Shaping ZnO nano-materials enhances their interactions with microorganisms, improves the penetration and disruption of cell membranes, and increases the release of antimicrobial agents.

## Figures and Tables

**Table 1:** Comparison of various types of materials for antimicrobial activity.

Material Type	Pros.	Cons.	Ref.
Silver-based materials	Broad-spectrum antimicrobial activity Long-lasting efficacy Low toxicity to human cells Intercepts biofilm formation	Toxicity issue at high concentrations Environmental concerns Limited efficacy against some microorganisms Incompatible with wound healing Uneconomical	[36,37]
Copper-based materials	Broad-spectrum antimicrobial action Durability and long lasting Fast microbial inactivation	Toxic at high concentrations Unpleasant odor and taste Regulatory issue	[39,40]
Quaternary ammonium compounds (QACs)	Broad-spectrum antimicrobial activity Anti-corrosion Long-standing residual activity Ease to apply	Potential for microbial resistance Limited activity Skin irritation problem Activity depends on environmental factors Less stable in elevated pH levels	[60,61]
Chitosan-based materials	Biocompatibility Biodegradability Adhesive and antioxidant properties Broad-spectrum antimicrobial effect	Poor solubility in neutral pH levels Fluctuating antimicrobial performance Chelation of metal ions pH dependent	[42,43]
Essential oils	Natural origin Aromatherapeutic behavior Broad-spectrum antimicrobial activity Eco-friendly Less likely of resistance	Fluctuating performance Allergies and sensitivity Volatility and evaporation Storage and shelf life Limited perseverance	[45,46,62]
Nano-materials	High surface area Easy penetration Targeted delivery Controlled release Less resistance development	Toxicity issue Limited understanding of prolonged activity Fabrication challenges Agglomeration problem Standardized testing protocol deficiency	[16,48]
Enzymes	Biological origin Biodegradability Low toxicity to humans Precision Responsive to environmental factors	Dependent on environmental conditions Limited spectrum of activity Sensitivity to processing conditions Limited shelf life	[50,51,52]
Polyhexanide-based materials	Low toxicity Broad-spectrum antimicrobial activity Against biofilm formation Long-lasting antimicrobial action Easy solubility	Limited activity Allergic issues Resistance issues Potential staining Environmental persistence	[53,54,55]
Clay minerals	Abundant and natural Broad-spectrum antimicrobial activity pH stability Non-toxicity Biocompatibility	Fluctuating performance Water solubility problem Potential environmental impact Particle aggregation Cytotoxicity issue Instability in unfavorable environment	[56,57]
Noble Gases	Inert nature Non-toxicity High diffusivity Broad-spectrum antimicrobial action	Uneconomical Unstable in composites Lack of clarity in mechanism Anesthesia issue at elevated levels Management problem Restricted penetration	[58,59]
Zinc-based materials	Broad-spectrum antimicrobial activity Low toxicity Biocompatibility Eco-friendly Less likely resistance	Limited solubility Fluctuating performance among microbes Probable accumulation in body	[12], This work

**Table 2:** Comparison of microbicidal and microbiostatic mechanisms.

Method	Pros.	Cons.
Microbicidal	Disease prevention. Microbicidals play a key role in preventing the spread of infectious diseases. They help eliminate or control microbes that can cause illnesses in humans. By killing or inhibiting the growth of harmful microbes, they can reduce the risk of infections [73]. Improve hygiene. Microbicidals are commonly used in cleaning and sanitization practices. They can effectively disinfect surfaces, equipment, and objects, promoting good hygiene. Hospitals, laboratories, food processing facilities, and public spaces pose special risks of contamination [74]. Health impact. Microbicidals have had a significant impact on public health by controlling the spread of infectious diseases. A number of pathogens have been reduced through their use, improving the health outcomes of people in general [75]. Versatility. Microbicidals come in various forms, including liquids, sprays, wipes, and gels. This versatility allows for easy application on different surfaces and in various contexts. They can be used on skin, medical equipment, household surfaces, and even in water treatment processes [76].	Resistance development. The overuse or misuse of microbicidals can contribute to the development of microbial resistance. Microorganisms have the potential to adapt and evolve, leading to a reduced effectiveness of certain microbicidal agents over time. This is a significant concern in healthcare sectors where multi-drug resistant bacteria can emerge [77]. Environmental impact. Some microbicidal compounds have adverse effects on the environment. When disposed of improperly or released into water bodies, they can contaminate ecosystems and impact aquatic life. Additionally, the production and disposal of microbicidals can contribute to pollution and waste generation [74]. Collateral damage. Microbicidals are designed to target and eliminate microbes, but they can also affect useful microbes. In some cases, the use of microbicidals can disrupt the natural balance of microbial communities, both externally on surfaces and internally in the human body. This can have implications for the overall health and functioning of ecosystems and humans [78]. Safety concerns. Certain microbicidals may pose health risks if used incorrectly or in excessive quantities. Some individuals may develop allergic reactions or skin irritations when exposed to certain microbicidal agents [76].
Microbiostatic	Preservation. Microbiostatics can be used in various industries, such as food and beverage, pharmaceuticals, and cosmetics, to preserve the quality (over the storage time) of products. By inhibiting microbial growth, they prevent spoilage, contamination, and the proliferation of harmful pathogens [79]. Selectivity. Microbiostatic agents can target specific types of microorganisms, allowing for selective control. This is beneficial when the goal is to inhibit the growth of harmful bacteria or fungi while leaving beneficial microorganisms unaffected [75]. Reduced resistance. Compared to microbicidals, microbiostatics are less likely to induce resistance in microbes. Since they do not kill the microbes outright, there is less selective pressure for the development of resistant strains. This can be advantageous in long-term (continuous) antimicrobial use [80]. Lower toxicity. Microbiostatic agents generally have a lower toxicity profile compared to microbicidal agents. This means they are less harmful to host cells and tissues and can be used at higher concentrations without significant adverse effects. This low toxicity makes them suitable for applications where direct contact with living organisms is required [81].	Limited effectiveness. Microbiostatic agents inhibit only microbial growth without killing them. This means they may not be effective in situations where the rapid and complete elimination of pathogens is pivotal. Microbes may continue to multiply once the concentration of the microbiostatic agent decreases or when conditions become more favorable for growth [79]. Potential rebound growth. When the inhibitory effect of a microbiostatic agent is removed, there is a risk of microbe rebound growth. If the conditions become favorable again, the microorganisms that were previously inhibited may resume their growth, leading to a resurgence of the microbial population. This rebound effect can pose challenges, especially where maintaining long-term control is needed [82]. Need for continuous application. Unlike microbiocidal agents that can provide a sustained antimicrobial effect, microbiostatic agents typically require a continuous supply to maintain their activity. Discontinuing the use of microbiostatics may allow microbial growth to resume. This can be a disadvantage when considering cost and convenience [83]. Interference. Microbiostatic agents can interfere with microbial diagnostic tests by inhibiting the growth of microbes. This interference may lead to wrong conclusions [84].

**Table 3:** Time course of ZnO-based materials for antimicrobial activity.

Activity	Time Course	Note	Ref.
Immediate contact and interaction	Initial minutes	When ZnO-based antimicrobials make contact with microbes, immediate interaction initiates. Adhesion and physical contact to microbial surfaces occur, and ZnO NPs start to release Zn^2+^ ions.	[12,120]
Early disruption of microbial membrane	Minutes–hours	ZnO NPs, especially in nano size, disrupt microbial membranes within the early hours. Involves the generation of reactive oxygen species (ROS), cell membrane damage, and interference with important cellular activities.	[12,121]
Zn^2+^ ion release and intracellular influence	Hours–days	In hours to days, ZnO-based materials continue to release Zn^2+^ ions, and they penetrate microbial cells, leading to DNA damage, enzyme inhibition, and interference with signaling activities.	[122,123]
Microbial growth inhibition	Days	As Zn^2+^ ions accumulate, the growth and reproduction of microbes are inhibited. ZnO-based materials exert a bacteriostatic or fungistatic effect, preventing the proliferation of microbes.	[124]
Continuous antimicrobial activity	Days–weeks	Antimicrobial action is sustained for prolonged time due to ZnO NPs. It involves maintaining the effective amount of Zn^2+^ ions or other active agents, prohibiting microbial expansion and biofilm formation.	[125]
Long-term residual effects	>Weeks	ZnO-based composites can produce residual effects, providing continuous antimicrobial activity for an extended period. Especially important in surface coatings.	[126]
Adaptation and resistance dynamics	Weeks–months	Microbes might develop adaptation to ZnO-based antimicrobials over time. Continuous exposure can lead to microbial strains with decreased susceptibility, spotlighting the relevance of continuous assessment.	[126]

**Table 6:** Characteristics of one-dimensional nanostructures of ZnO-based materials and their microbiostatic activities.

Material	Target Microbe	Time Temp	Synthesis Method	Note	Ref.
ZnO NW@PP ^a^	*E. coli* *B. subtilis*	24 h, 37 °C	Chemical bath deposition	An antimicrobial test was performed with the presence of fluorescent light. Clear growth inhibition was observed for *B. subtilis*, but almost not for *E. coli*. A plasma treatment was used before the chemical bath deposition.	[173]
ZnO NR ^b^	*S. aureus* *B. subtilis* *E. coli* *A. aerogenes*		Hydrothermal	The rods have an average diameter and length of 45 and 250 nm, respectively. In the presence of different concentrations of ZnO NR, *S. aureus* and *B. subtilis* did not show any growth even at a lower concentration of 100 μg/mL. For *E. coli* and *A. aerogenes*, a 500 μg/mL concentration was enough for inhibition observation.	[174]
Ti-ZnO NRS ^c^	*E. coli* *S. aureus*	24 h, 37 °C	Hydrothermal	Different ZnOs produced good long-term antibacterial effects and a poor short-term antibacterial effect with E. coli, due to the weak bacteriostatic property of ZnO against *E. coli*. Antibacterial effect: due to the rapid release of ZnO nanospheres.	[175]
ZnO-rod ZnO-plate	*E. coli* *S. aureus*	24 h, 37 °C	Combustion, O_2_ annealing	Nano-sized/one-dimensional rod and nano-sized/one-dimensional plate ZnO were prepared from commercial ZnO (bulk). Nano-sized/one-dimensional rod: diameter = 30 to 180 nm and length = 100 to 300 nm. Nano-sized/one-dimensional plate: width = 40 to 250 nm and length = 80 to 350 nm. One-dimensional ZnO size = 30 nm to 300 nm. Oxygen-annealed ZnO showed slightly higher antimicrobial activity than the unannealed ZnO against the target strains. After UV irradiation, the antimicrobial activities of the oxygen-treated materials increased by around 19%.	[176]

^a^ ZnO-nanowires@polypropylene membrane, ^b^ ZnO nanorods, ^c^ Titanium–ZnO nanorod–nanosphere hierarchical structure.
